# Brain Somatic Mutation in Aging and Alzheimer’s Disease

**DOI:** 10.1146/annurev-genom-121520-081242

**Published:** 2021-05-12

**Authors:** Michael B. Miller, Hannah C. Reed, Christopher A. Walsh

**Affiliations:** 1 Division of Genetics and Genomics, Boston Children’s Hospital, Boston, Massachusetts 02115, USA;; 2 Department of Pediatrics, Harvard Medical School, Boston, Massachusetts 02115, USA; 3 Division of Neuropathology, Department of Pathology, Brigham and Women’s Hospital, Harvard Medical School, Boston, Massachusetts 02115, USA;; 4 Broad Institute of MIT and Harvard, Cambridge, Massachusetts 02142, USA; 5 Allegheny College, Meadville, Pennsylvania 16335, USA;; 6 Howard Hughes Medical Institute, Boston, Massachusetts 02115, USA; 7 Department of Neurology, Harvard Medical School, Boston, Massachusetts 02115, USA

**Keywords:** somatic mutation, Alzheimer’s disease, mosaicism, neurodegenerative diseases, genomics, aging

## Abstract

Somatic mutations arise postzygotically, producing genetic differences between cells in an organism. Well established as a driver of cancer, somatic mutations also exist in nonneoplastic cells, including in the brain. Technological advances in nucleic acid sequencing have enabled recent break-throughs that illuminate the roles of somatic mutations in aging and degenerative diseases of the brain. Somatic mutations accumulate during aging in human neurons, a process termed genosenium. A number of recent studies have examined somatic mutations in Alzheimer’s disease (AD), primarily from the perspective of genes causing familial AD. We have also gained new information on genome-wide mutations, providing insights into the cellular events driving somatic mutation and cellular dysfunction. This review highlights recent concepts, methods, and findings in the progress to understand the role of brain somatic mutation in aging and AD.

## INTRODUCTION

Somatic mutations are acquired alterations in the genome of an organism. Unlike germline mutations, which are typically inherited from a parent and are present throughout the body, somatic mutations arise in an organism after the single-cell zygote state and exist only in a subset of the organism’s cells. Distinct classes of somatic mutation include single-nucleotide variants (SNVs); aneuploidy; copy number variants, including insertions and deletions; structural variants; and transposable element (TE) insertions. Somatic mutations have been studied for decades in the context of neoplasms, where they form the foundational theory of carcinogenesis (41, 113, 124). More recently, somatic mutations have also been identified in nonneoplastic tissues (44, 77, 78), including the brain (48, 75), raising interest in a potential role in the brain during aging and age-related neurodegenerative diseases. Here, we review recent developments in the occurrence and potential mechanistic roles of somatic mutations in the brain during aging and Alzheimer’s disease (AD). We focus on brain somatic SNVs in these conditions, with a discussion of normal neuronal somatic mutation and AD genetics, and through this lens we examine somatic SNVs in other neurodegenerative contexts as well as the spectrum of other classes of somatic mutation in AD.

## DISTRIBUTION AND DETECTION OF SOMATIC MUTATIONS

**Figure 1:**
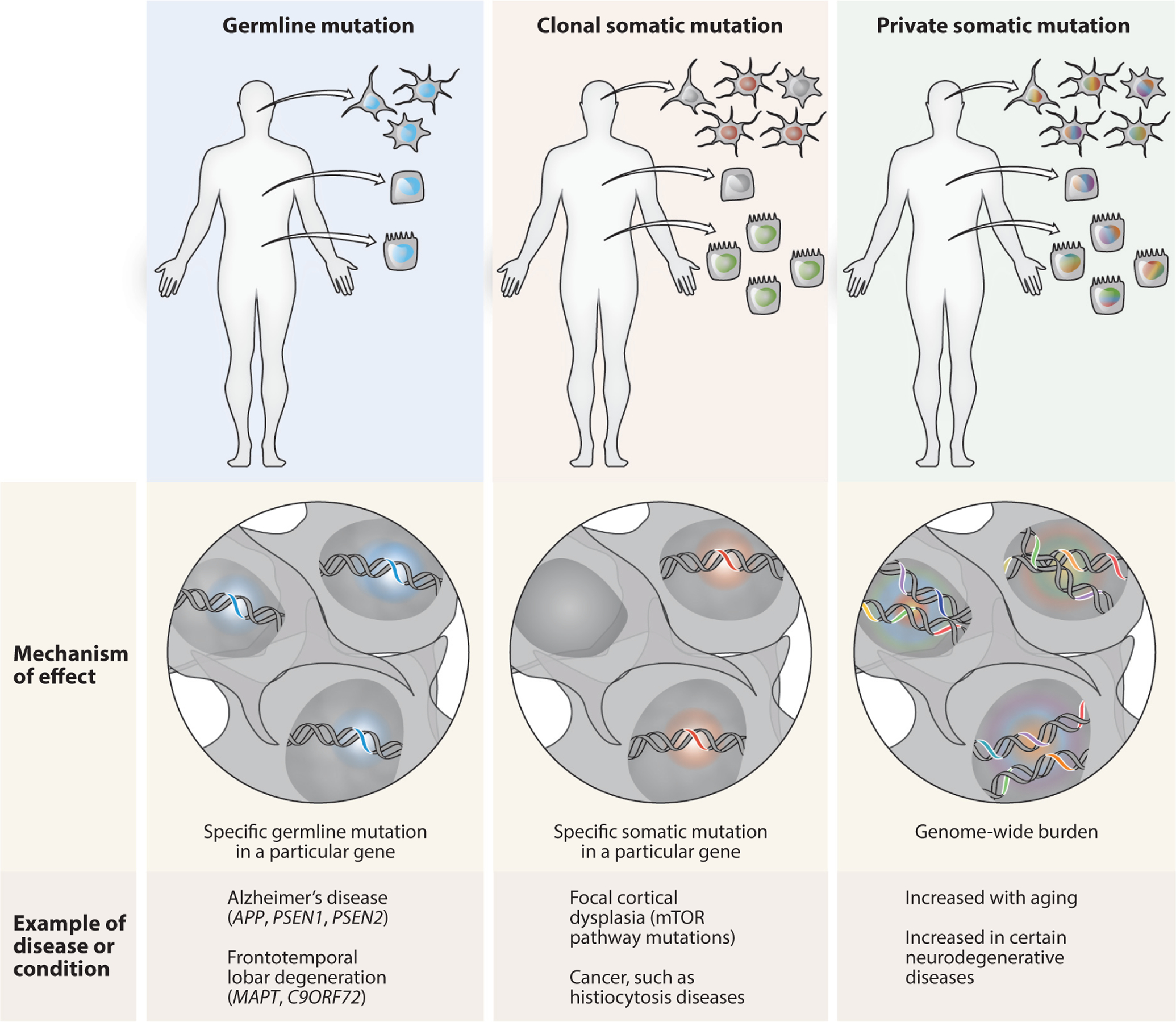
Distribution of germline and somatic mutations. Genetic mutations can occur in the germline or in a somatic subset of cells. Germline mutations (*blue*), whether inherited or occurring de novo, are found in every cell in the body. In relevant cell types, germline mutations cause classical genetic diseases, which in the brain include familial Alzheimer’s disease and other familial neurodegenerative diseases. Clonal somatic mutations arise during development or other proliferative conditions, and are found in all daughter cells but not elsewhere. Clonal somatic mutations can define distinct subpopulations of cells within a tissue, such as a group of neurons (*red*) or a clone elsewhere in the body (*green*). Clonal somatic mutations can cause diseases ranging from cancers to focal cortical dysplasia, typically related to the specific mutated gene and cell type. Private somatic mutations (*multiple colors*) occur in individual postmitotic cells such as brain neurons, are unique to that cell, and increase in aging and certain neurodegenerative diseases. Rather than acting through a single specific gene, private somatic mutations are noteworthy in their total mutational burden and broad potential genomic effects. Figure created by Ken Probst of Xavier Studio, with input from the authors.

In contrast to germline mutations, somatic mutations are present only in a subset of cells, forming a mosaic where the organism has a heterogenous composition of cellular genomes. Somatic mutations may occur in multiple cells (clonal) or may be unique to a single cell (private), depending on the mechanism and timing of their occurrence (Figure 1).

**Figure 2:**
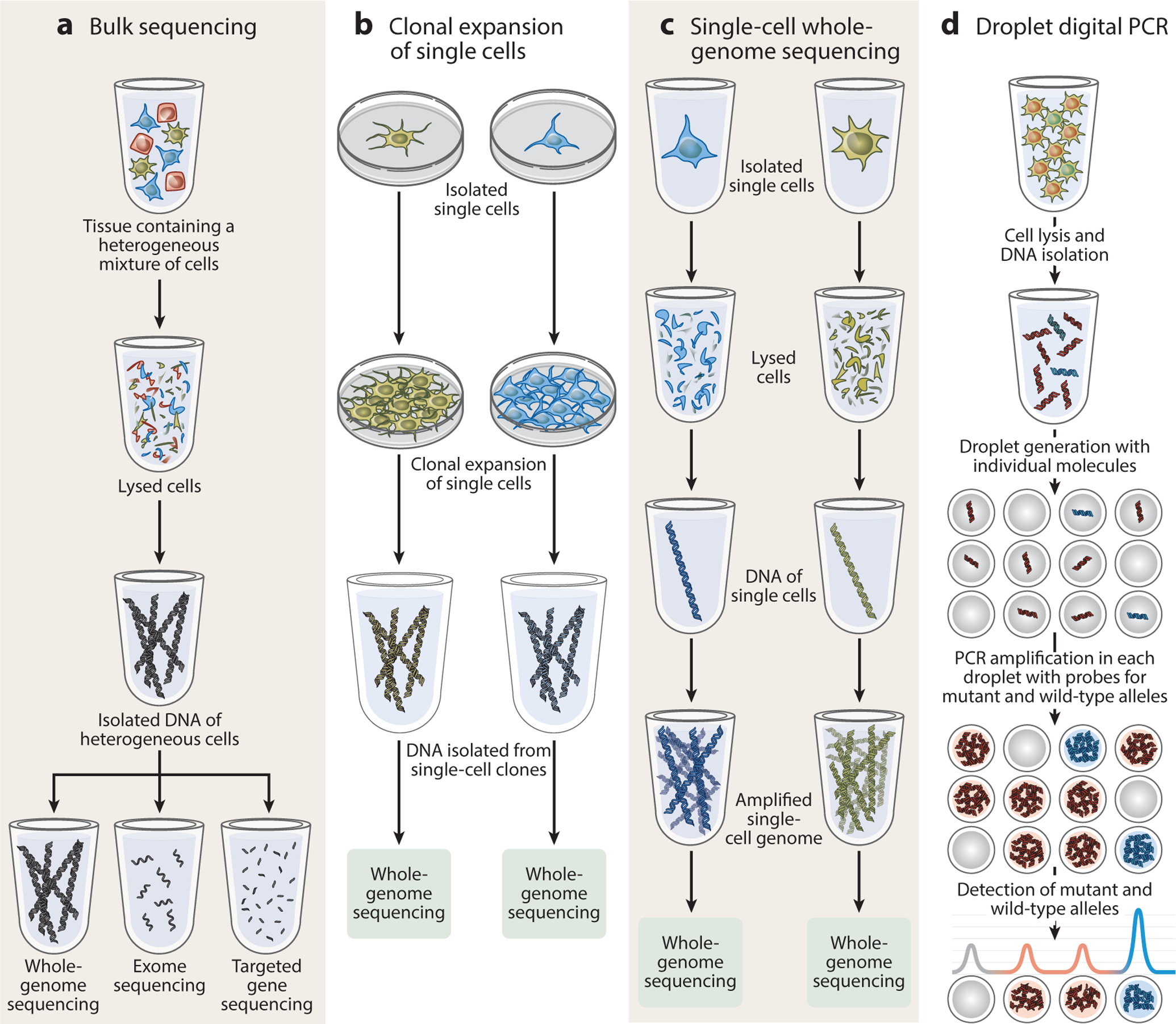
Examples of methods for detecting somatic mutations in bulk and single cells. The methods illustrated here focus on detecting somatic single-nucleotide variants, though some can also identify copy number variants and other classes of somatic mutation. (*a*) Bulk sequencing can be used to detect clonal somatic mutations, as they are present in multiple cells in a sample. DNA is isolated from a heterogeneous sample of cells, then used to prepare libraries for whole-genome sequencing or targeted sequencing methods, such as the exome or a specific set of genes. Bulk methods are used to detect variants arising during development, physiological proliferation, or neoplasia. Bulk sequencing is not well suited to detecting private mutations that are exclusive to individual cells, which generally require single-cell detection methods. However, bulk methods have higher throughput than current single-cell DNA sequencing methods, conveying information about more cells per experiment. (*b*) Clonal expansion of single cells in culture enables the biological amplification of the genome of a single initial cell, generating enough genome copies for detection by sequencing methods. Clonal expansion can be performed with natively proliferating cells or embryonic stem cells derived from nuclear transfer of any cell type. This method is susceptible to cell culture artifacts but minimizes in vitro genome amplification concerns. (*c*) Single-cell whole-genome sequencing facilitates the direct examination of the genome of a single target cell, typically using in vitro enzymatic genome amplification (e.g., by multiple displacement amplification). This technique can be used on any cell type (or even naked nuclei) that can be isolated using fluorescence-activated cell sorting or similar methods, and therefore can be applied to nondividing cells. (*d*) Variant-specific detection methods include droplet digital PCR. Bulk DNA is mixed with variant-specific primers and fluorescent probes, then fractionated into droplets. PCR amplification occurs on individual DNA fragments to produce a binary signal for each droplet and thus a quantitative measure of the variant allele fraction, and therefore this approach is often more sensitive than bulk sequencing methods. Figure created by Ken Probst of Xavier Studio, with input from the authors.

When arising in proliferating cells, somatic mutations take a clonal pattern, with all daughter cells exhibiting the same mutation. In the context of development, a new somatic mutation would be passed on to all daughter cells, delineating a clone of that lineage. Depending on the developmental timing of the mutation, a new somatic variant may be widely distributed in multiple tissues, or may be restricted to a single tissue or even a narrower localization. In the context of neoplasia, a somatically mutated cell can have a selective growth advantage and proliferate to create a clonal population. Both developmental and neoplastic clonal somatic mutations are thus present in multiple cells and can generally be detected using bulk DNA sequencing methods, whether using whole-genome or targeted methods (Figure 2).

When a cell specializes and is no longer undergoing cell division, it is said to be postmitotic. Surprisingly, at least some of these noncycling cells also appear to acquire somatic mutations, which are exclusive or private to that individual cell. Since these variants are not clonally expanded, they are not detectable using traditional DNA sequencing methods and require specialized methods for single-cell detection. These single-cell methods may also be applied to proliferating cell populations to define the genomic properties of individual cells. Somatic mutations may also be detected by variant-specific methods such as droplet digital PCR (120, 133). Recent advances in molecular biology and informatics have also paved the way for the detection of somatic variants in expressed RNA transcripts, by bulk or single-cell approaches, though significant hurdles exist to identify mutations from transcripts, including uneven coverage along each transcript’s length and between genes (33, 40, 53, 85, 95).

## SOMATIC MUTATIONS IN THE BRAIN

Somatic SNVs accumulate during human brain development, with an estimated 200–400 somatic SNVs already present per cell at mid-gestation (8). Mutations acquired during development may be functionally silent, while serving to identify cells descended from the same progenitor for lineage tracing (75). If such mutations alter cellular physiology, they can alter tissue structure and function and result in developmental neurological disorders (45, 55, 97). For example, pathogenic somatic mutations in mTOR pathway genes in certain brain progenitors result in hemimegalencephaly (66, 96, 105), and similar mutations in a more limited distribution produce focal cortical dysplasia (9, 10, 26, 27). Somatic mutations may also directly affect the electrical physiology of neurons, as the expression of the *Braf* V600E variant in mouse neuronal progenitors contributes to epileptogenicity (64). Somatic mutations have also enabled studies tracing the origin of cancers—for example, providing evidence that glioblastoma tumors share somatic mutations with subventricular zone progenitor cells, their potential cellular origin (67).

## SOMATIC MUTATIONS AND SIGNATURES IN AGING

To study aging, experimental approaches such as single-cell whole-genome sequencing (scWGS) (37, 73, 132), both in vitro (12, 36, 90) and in vivo clonal expansion (70), and dilution bottleneck sampling sequencing (51) have revealed various rates of somatic mutation accumulation in different tissue types. scWGS, which allows the detection of private mutations present in individual cells, has revealed numerous neuronal somatic SNVs present per cell even in neurotypical individuals (75). Somatic mutations have been identified as increasing in neurons during the course of human aging (74). In neurons, somatic SNV levels rise with age at a rate of approximately 20 new mutations per year, a concept known as genosenium that reveals novel insights about the aging process (73). Analysis of the specific DNA base changes and their trinucleotide contexts can identify signatures that reflect the origin of those somatic mutations (3, 4, 65).

Cancer genome analyses have identified a number of mutational signatures (3). Notably, Catalogue of Somatic Mutations in Cancer (COSMIC) signatures 1 and 5 (analogous to the single-base signatures SBS1 and SBS5 in the most recent version, COSMIC v3) were identified in tumor genomes as increasing with age in a clock-like manner, such that the abundance of these signatures corresponds to the age of an individual (2). Signature 1 contains predominantly C>T mutations, while signature 5 contains primarily C>T and T>C mutations (2). scWGS of 161 neurons derived from healthy and prematurely aging brains revealed a mutational signature, named signature A, that resembled signature 5 and correlated with age (73). A subsequent study using bulk exome sequencing also found an abundance of signature 5 in aged brain samples (93). While this study was not able to detect the full extent of mutations that can be found with single-cell experiments, it is noteworthy that the likely clonal somatic mutations detectable in bulk exome sequencing also showed aging-associated mutational signature 5 in the brain. Indeed, the aging-associated mutational signatures observed in the brain are similar to those seen in other tissues (Table 1).

## ALZHEIMER’S DISEASE GERMLINE GENETICS

Landmark studies of rare kindreds with forms of familial AD (FAD) have revealed several genes showing autosomal dominant genetic inheritance. In turn, these genes have illuminated biochemical mechanisms of protein processing and folding that constitute the basis of the major paradigm for AD initiation. Germline mutations in the amyloid-β (Aβ) precursor protein gene (*APP*) produce FAD (7, 46, 118), generally acting by increasing the quantity of Aβ peptide, which aggregates in Aβ plaques, a pathological hallmark of AD. Mutations in presenilin 1 (*PSEN1*) (109, 111, 112) and presenilin 2 (*PSEN2*) (72, 106) also cause FAD, as these genes encode components of the γ-secretase enzyme complex, which cleaves the APP protein and, in conjunction with β-secretase proteolytic cleavage, produces Aβ peptide. Each of these FAD genes has the effect of increasing Aβ, which forms small misfolded oligomer aggregates that damage neurons and induce hyperphosphorylation of tau (56, 110), another pathologic hallmark of AD.

Fully penetrant genetic causes of AD only account for less than 1% of cases. Linkage studies and subsequent genome-wide association studies have identified many genes with increased risk for AD. For late-onset AD, which is significantly more common than the early-onset AD caused by most FAD variants, the *APOE* ε4 allele confers the greatest risk, 80% lifetime for homozygotes (57, 94, 114). These broad genomic approaches have identified *TREM2* and a large number of other genes that also confer some risk of AD (23).

## SOMATIC MUTATIONS AS A POTENTIAL CAUSE OF ALZHEIMER’S DISEASE

While germline mutations in the genes *APP*, *PSEN1*, and *PSEN2* are known to cause early-onset familial AD, these mutations account for only a small fraction of cases, as the majority of individuals with AD develop the disease without a fully penetrant genetic cause (57). Such nonfamilial AD (also referred to as sporadic or non-Mendelian AD) often arises later in life than familial AD and thus significantly overlaps with late-onset AD. Therefore, it has been hypothesized that somatic mutations in familial AD genes may cause late-onset AD, with the lower cell fraction or limited spatial distribution of mosaic mutations serving to explain the later onset of disease. In such a case, misfolded proteins first generated from a sparse somatic mutation might spread to other areas of the brain by means of templated protein misfolding (14), in a similar manner as occurs during the spread and misfolding of prions (13, 83, 84). Indeed, both Aβ and tau have shown such templated misfolding in various systems (25, 60, 86), which has led investigators to examine several classes of somatic mutation for a potential role in late-onset AD pathogenesis (100).

## SOMATIC SINGLE-NUCLEOTIDE VARIANTS IN ALZHEIMER’S DISEASE, FOCUSING ON FAMILIAL ALZHEIMER’S DISEASE GENES

Given the significant role of germline SNVs in familial AD, studies of somatic mutation in AD have focused largely on SNVs. An examination of late-onset AD cases for mosaic variants in *APP*, *PSEN1*, and *PSEN2* using single-gene Sanger sequencing methods found no somatic mutations (104). Beck et al. (11) reported an AD case with *PSEN1* somatic mosaicism present in 14% of cells in the cerebral cortex and in 8% of peripheral lymphocytes. This level of *PSEN1* mosaicism thus appears to be capable of contributing to disease, perhaps with a delayed onset relative to germline mutation. Notably, the pathogenic allele was also present in the individual’s germ cells, as her daughter had inherited the mutant allele and subsequently presented with early-onset AD, at the age of 27.

However, subsequent studies with more advanced sequencing methods and greater case numbers have not provided additional examples to strongly support the somatic mutation Aβ initiation hypothesis beyond Beck et al.’s (11) *PSEN1* case report. Multiple recent studies have utilized targeted deep next-generation gene panel sequencing approaches and have found very few somatic mutations in *APP*, *PSEN1*, and *PSEN2* in AD brain. One such study applied targeted sequencing to 72 AD cases and 58 non-AD controls, demonstrating a sensitivity of detection of somatic SNVs down to a 1% variant allele fraction (108). This study identified two somatic mutations in the tau-encoding *MAPT* gene within AD entorhinal cortex and one *PSEN2* somatic variant in a control subject but did not observe any somatic variants in familial AD genes in subjects with AD. A similar study examined brain tissue in 100 AD and 52 control subjects, with no pathogenic somatic variants in *APP*, *PSEN1*, *PSEN2*, or other targeted genes, though additional blood samples did show scattered somatic variants, including two potentially damaging mutations in *SORL1* (87). Whole-exome sequencing of hippocampus in 17 AD cases found no pathogenic mutations in *APP*, *PSEN1*, or *PSEN2* (92). Similar results were seen in another exome study of a larger cohort of 244 AD cases, which found 22 somatic variants with a high variant allele fraction (>10%) but none in FAD genes, across a group of 1,461 humans with various neurodegenerative diseases (125).

Park et al. (93) went further, performing whole-exome sequencing on tissue microdissected from multiple hippocampal subregions from 52 AD cases and 11 controls, and found no pathogenic somatic mutations in familial AD genes. Another recent study performed SureSelect targeted sequencing of 102 genes on multiple brain regions from 20 AD cases, 20 Lewy body disease cases, and 14 controls, reporting minimal mutations in neurodegeneration-associated genes in all groups (58). Focusing on the temporal cortex, another study performed targeted sequencing of AD-related genes from 8 AD cases and 8 controls, which showed no somatic instances of AD-associated mutations but did report a *CD55* regulatory region variant in one case (50). Efforts have also been made to identify somatic variants in AD using RNA sequencing data, including a potential AD role for the intellectual disability–related *ADNP* gene (53), though this exciting and novel approach is constrained by difficulty in distinguishing somatic variants from germline ones and also in excluding RNA editing, which can be misinterpreted as somatic mutation.

Taken together, these numerous studies have not shown somatic SNV mutation of FAD genes to be a significant mechanism in the pathogenesis of late-onset AD, nor have studies found that somatic variants in familial genes play a significant role in other neurodegenerative disorders, such as Parkinson’s disease (71, 101, 102). These studies have been limited by detection sensitivity (generally not below a 1% variant allele fraction), and therefore further investigation with novel ultrasensitive approaches may provide new insights (30). Furthermore, the death of single or rare mutated cells might prevent detection altogether, making it difficult to discount the disease-initiating mutation hypothesis entirely.

## SOMATIC ANEUPLOIDY AND COPY NUMBER VARIATION IN ALZHEIMER’S DISEASE

Somatic changes in the number of copies of individual genes and whole chromosomes have been reported in human brain cells (18, 63, 81, 103, 130), raising the question of whether such somatic copy number changes play a role in AD. For reference, aneuploidy is used to describe full chromosomal copy changes, and the term copy number variant is generally applied to changes greater than ~ 1 kb in size but smaller than the ~5-Mb karyotyping resolution, and these events are sometimes detected as DNA content variation. Copy number studies in AD have focused largely on chromosome 21 and the *APP* locus specifically, because germline trisomy 21 (Down’s syndrome) increases the lifetime risk of AD, largely through *APP* overexpression (49). These studies have yielded some-what contradictory results, generating uncertainty regarding the possible role of somatic *APP* copy number variants in AD.

A case of early-onset dementia with 10% mosaic trisomy 21 (107) prompted the hypothesis that trisomy 21 mosaicism may cause nonfamilial AD (98). Using fluorescence in situ hybridization, studies on AD have found mosaic trisomy and other aneuploidies of chromosome 21 in various cell types, including fibroblasts (43), buccal epithelial cells (119), and neurons and other brain cells (52). However, one of these studies noted no difference in trisomy 21 rates between AD hippocampal cells and controls (119). Examining potential aneuploidy at greater cytogenetic resolution than full chromosomal gains, another study reported focal *APP* gains in the majority of AD neurons—up to 12 copies, with a mean of 3.8 copies per AD neuron, compared with 1.6 copies in control neurons, based on quantitative PCR analysis (16). By contrast, a whole-exome sequencing experiment using brain DNA from 289 AD cases did not confirm this claimed increase in *APP* copy number, identifying only a single individual to have *APP* triplication, which was interpreted as actually representing a potential germline event (59). Using low-coverage scWGS, another group found very low levels of aneuploidy, with no selective gain of chromosome 21 in AD patients (122). This study also reported no focal *APP* copy number gains, though the sequencing coverage may not have been sufficient to rule out single-gene events.

Observations of potential copy number changes involving the *APP* gene, although not confirmed by all groups, led to the examination of mechanisms that could produce such somatic changes at that locus. Lee et al. (69) reported—in AD and control neurons—somatic copy number gains of *APP* genes lacking introns, terming them genomic cDNAs (gencDNAs). However, Kim et al. (62) did not observe such events in scWGS data from single AD and control neurons, which should have been well powered to detect them. Independent analysis of the Lee et al. (69) hybrid capture sequencing data by Kim et al. (62) subsequently revealed that the data were contaminated by a plasmid vector with an *APP* gene insert, at a level of abundance sufficient to account for the full *APP* cDNA signal, raising doubt about the gencDNA report. Kim et al. (62) also found that sequences resembling proposed gencDNA, which have been reported elsewhere (93), may be derived from normal mRNA and PCR-generated nested sequences. Lee et al. (68) subsequently reported observing gencDNA in additional experiments, after reducing the potential for vector contamination. Further studies and analyses from independent research groups would therefore be of value to clarify the basis of this observation and its potential relevance for AD biology.

Conflicting reports also exist on somatic copy number changes in chromosomes other than 21 in AD. In AD brains, several studies have found an increased incidence of aneuploidy compared with that observed in nondiseased individuals (5, 6, 131), while others report no detectable difference in various copy number comparisons between AD cases and nondiseased controls (108, 117, 122, 127). Outside the brain, in blood cells, a prospective study revealed that men with increased somatic loss of the Y chromosome were more likely to be diagnosed with AD, with a hazard ratio (6.8) higher than even that of the *APOE* genotype (2.8) (32). While there remains significant debate over potential copy number changes in AD, these reports represent intriguing potential mechanisms in disease.

## SOMATIC TRANSPOSABLE ELEMENT MOBILIZATION IN ALZHEIMER’S DISEASE

TEs are mobile DNA sequences that can change location within the genome and include classic transposons (class II TEs, the so-called jumping genes) that operate via a cut-and-paste mechanism, as well as retrotransposons (also known as retroelements and class I TEs) that utilize an RNA intermediate and reverse transcriptase in a copy-and-paste mechanism (15). In humans, retroelements account for nearly half the genome; they are dominated by inactive sequences but have a small active subset. Somatic retrotransposition has been documented in human neurons (34, 35, 121). Retrotransposons have been hypothesized to play a role in age-related neurodegenerative diseases, including AD and frontotemporal degeneration–amyotrophic lateral sclerosis (89).

Studies in *Drosophila* models of tauopathy have revealed that tau pathology is associated with increased chromatin relaxation (38). A pair of studies also showed dysregulated transcription of TEs in these fly models, along with increased expression of some TEs in human tauopathy diseases (47, 116). However, there is no overlap between the TEs activated in AD in the two papers, making it difficult to draw firm conclusions about TE activation in these AD models. Furthermore, while each of these studies evaluated TE transcription, less is known about somatic TE insertion rates in the DNA in AD. One study reported finding no difference in LINE1 copy number in bulk brain and blood between AD patients and controls, and no relationship between age and LINE1 copy number (99). Direct examination for somatic retrotransposition at the single-cell level in AD may clarify the extent of this phenomenon and the proposed relationship with disease mechanisms.

## SOMATIC MUTATIONS IN CANCER GENES AND IMPLICATIONS FOR NEURODEGENERATION

While much of the attention on somatic mutations in AD and other neurodegenerative diseases has focused on a somatic version of familial disease genetics, clues from other disorders suggest that multiple somatic mechanisms may produce neurodegeneration. For example, certain neurodegenerative phenotypes can occur in patients with the somatic mutation–driven neoplasm Langerhans cell histiocytosis (128), which results from the proliferation of myeloid cell precursors (22), often driven by *BRAF* V600E and other MAPK pathway variants. In such individuals, lesions occur in the cerebellum and basal ganglia, with corresponding clinical neurological symptoms. To further investigate the possible role of somatic mutations and histiocytosis in neurodegeneration, Mass et al. (79) developed mice expressing *Braf* V600E in specific yolk sac erythro-myeloid progenitors that populate the brain in early development and generate microglia, the brain tissue-resident macrophages. These mice showed clonal expansion of tissue-resident macrophages and severe late-onset neurodegenerative disease, bolstering the link between somatic mutation–driven proliferation and neurodegeneration. Indeed, a diverse group of somatic variants can cause histiocytosis diseases (28), providing a variety of potential genes that could lead to neuronal dysfunction in a similar manner as in Langerhans cell histiocytosis. Whereas limited studies have so far not revealed *BRAF* V600E mutations in AD brain (58, 93), small numbers of cases show mutations in *DNMT3A* or *TET2*, which are cancer-associated genes that are also mutated in clonal hematopoiesis (44, 54), or in the PI3K, MAPK, or AMPK pathways, whose significance must be evaluated through studies of larger sample size. These investigations have only begun to evaluate potential relationships among cancer-associated genes, cellular proliferation, and neurodegeneration.

## GENOME-WIDE SOMATIC MUTATIONS

Beyond the effect of variants in a single gene, the full aggregate of somatic mutations in the genome carries the potential to significantly impact cellular function and health. While sequencing technology has developed dramatically in recent years (123), the majority of studies are performed on bulk tissue and are thus best suited to detecting variants present in multiple clonal cells, as discussed above. Bulk approaches are generally unable to detect private mutations in individual cells, which limits the inferences that can be made from negative results, and indeed current studies generate conflicting conclusions. Some bulk sequencing studies have suggested that there is no significant difference in somatic SNV count in the brains of AD and non-AD control individuals (58, 93), in contrast to a report of somatic mutations being uniquely present in AD brains and absent in controls in targeted sequencing data (50).

Single-cell methods are able to detect mutations that are present only in individual cells (20, 31, 35, 129, 134), which indeed may make up the majority of a neuron’s somatic mutation burden (75). These single-cell mutations appear to be present in the hundreds at birth (8, 73) but then, remarkably, increase at a rate of approximately 20 SNVs per year, leaving neurons with thousands of such somatic SNVs in old age. In individuals with a neurodegenerative phenotype linked to deficient nucleotide excision repair (NER), manifesting as Cockayne syndrome or xeroderma pigmentosum, scWGS on neurons revealed a significant increase in somatic SNVs compared with normal neurons (73). This observation raises the possibility that a genome-wide increase in neuronal somatic mutations may also occur in other neurodegenerative diseases. The somatic SNVs in NER-deficient neurons do not fall in a single gene or genomic area, but instead are broadly distributed across the genome, in a similar manner as somatic SNVs acquired during the aging process (73). Furthermore, the somatic mutations in NER-deficient neurons showed a distinct composition of mutational signature patterns compared with controls.

## MECHANISMS OF SOMATIC MUTATION IN NEURODEGENERATION

**Figure 3:**
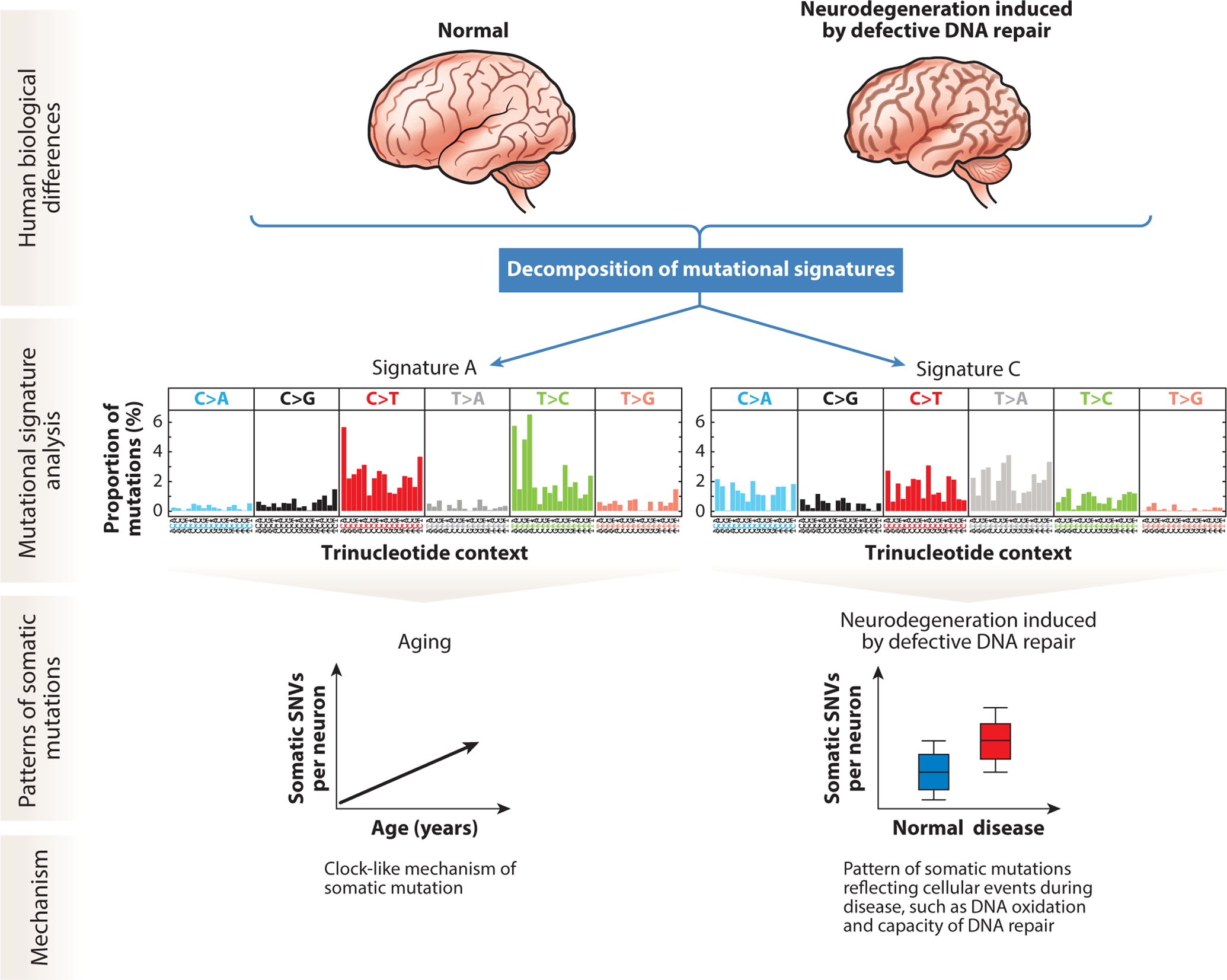
Neuron mutational signature analysis in aging and in neurodegeneration induced by defective DNA repair. Analysis of the specific base changes and their trinucleotide context reveals specific patterns of DNA alterations that are used to identify the causes of somatic mutations. Each cell’s mutational profile of the six possible base change types, subdivided by the 16 trinucleotide context combinations of 5′and 3′base identity, is used to computationally decompose signatures that align with biological variables in the samples. Signatures present in a given cell can point to biological events responsible for mutagenic forces that acted on that cell’s genome. Signature A, composed predominantly of C>T and T>C base changes, accumulates with age in neurons in a clock-like mechanism. Signature C, which contains multiple base changes, including C>A and T>A variants, is enriched in neurons in neurodegenerative diseases of NER deficiency. Signature C suggests oxidative damage and other events that surpass DNA repair capacity and lead to permanent DNA changes. Abbreviations: NER, nucleotide excision repair; SNV, single-nucleotide variant. Figure created by Ken Probst of Xavier Studio, with input from the authors.

Mutational signature analysis of scWGS data from NER-deficient neurons showed an abundance of signature C above the levels seen in control neurons (73). Signature C and the overall mutational profile in NER-deficient neurons point to specific mutagens and cellular processes that influence somatic mutation in these cells, and may act more broadly in neurodegeneration (Figure 3). Signature C contains C>A mutations, which are associated with oxidative damage to DNA in the form of 8-oxo-guanine and other altered bases (65), a result of reactive oxygen species produced during cellular metabolism. Indeed, oxidative damage has been previously identified in AD brain tissue (21, 39, 76, 82). Interestingly, exome sequencing of the hippocampus in AD also identified an oxidative mutational signature, more than half of which consisted of C>A mutations (93), whose detection by bulk sequencing indicates that they may potentially arise in a different manner than the predominantly private mutations identified in single cells. Increased oxidative DNA damage and reduced histone deacetylase HDAC1 activity were observed in transgenic mice expressing five germline AD-linked mutations (88), and this increase in oxidative damage is also observed in HDAC1-deficient mice (91), suggesting a link between chromatin structure and DNA damage (38), which may in turn lead to increased somatic mutations.

The observation of signature C mutations in human neurons that are genetically deficient in NER (73) indicates the involvement of NER in repairing lesions that lead to signature C somatic mutations. Therefore, somatic mutations may result from increased oxidative damage that accumulates beyond the capacity for NER and other DNA damage repair pathways to correct the DNA lesions. Furthermore, there is evidence linking AD-associated misfolding of tau (24, 29, 61) and Aβ (17) to DNA damage, potentially involving a toxic feed-forward loop between these mechanisms (1, 115).

## POTENTIAL EFFECTS OF ABUNDANT GENOMIC SOMATIC MUTATIONS IN NEURODEGENERATION

The DNA damage theory of aging postulates that DNA damage contributes to genomic instability and the overall process of aging (80). Somatic mutations indeed accumulate in neurons during typical aging (73), and more so in neurodegeneration from NER deficiency (73). How might these mutations lead to dysfunction in cells? These neurons show more nonsynonymous mutations, which change the encoded amino acid, and stop-gain mutations, which create a new stop codon that truncates protein translation. These changes can impair the function of processes that rely on full dosage of particular genes. Also, as mutations accumulate, this accumulation produces exponential increases in the proportion of cells that have biallelic inactivation, with modeling showing such an increase of so-called knockout neurons (73). The increase in nonsynonymous mutations also leads to a projected increase in neoantigen peptides that are produced in the cell and then presented by major histocompatibility complex (MHC) class I molecules to CD8+ T lymphocytes for immune surveillance. While it seems implausible for neoantigens from an individual cell to affect the broader immune response, clonal CD8+ T cells have been reported recently in AD brain (42), raising the possibility that this response may be related to intracellular events such as somatic mutation. Whether from gain or loss of function, somatic mutation accumulation stands to affect individual genes and the broader genome, which can play a role in cellular dysfunction and potentially cell death.

## CONCLUSIONS

Somatic mosaicism in the brain can range from many clonal cells to a private mutation in an individual cell. Specific clonal somatic SNVs cause neurodevelopmental diseases and may play a role in AD and other neurodegenerative diseases, though currently published studies have not established a clear role for somatic FAD gene mutations in causing AD. However, genome-wide, SNVs are increased in aging and in nucleotide repair deficiency–related neurodegeneration, where mutational signature analysis has enabled the identification of specific cellular processes involved in the generation of somatic mutations, which is of potential importance to broader cellular function in these conditions and neurodegeneration broadly. Furthermore, somatic mutations across the genome may lead to cellular harm through multiple mechanisms. Technological advances have enabled rapid progress in understanding somatic mutations in the brain, and future advances hold great promise to enable the detection of mutations at lower abundance, with higher cellular throughput for more complete analysis, and even accompanied by other cellular information (19), such as gene expression, chromatin structure, and protein misfolding.

## Figures and Tables

**Table 1:** Studies of somatic single-nucleotide variant signatures in the brain in aging and neurodegeneration, along with selected other human tissues

Study	Tissue/cell	Method	Biological context	Mutational signature(s)^a^
**Brain**				
Hoang et al. (51)	Bulk brain (frontal cortex), colon, kidney	Dilution followed by bulk whole-genome sequencing (BotSeqS)	Aging	None identified
Park et al. (93)	Bulk brain (hippocampus)	Bulk whole-exome sequencing	Alzheimer’s disease	COSMIC signature SBS18
Lodato et al. (73)	Neurons (prefrontal cortex)	Single-cell whole-genome sequencing	Aging	COSMIC signature 5
			DNA repair deficiency neurodegeneration	COSMIC signature 8
**Other tissues**				
Blokzijl et al. (12)	Adult stem cells of small intestine, colon, liver	Whole-genome sequencing of clonal organoid cultures derived from primary multipotent cells	Aging	COSMIC signature 5
Osorio et al. (90)	Hematopoietic stem cells	Whole-genome sequencing of clonal cultures	Aging	COSMIC signature 5
Franco et al. (37)	Skeletal muscle resident progenitor/stem (satellite) cells	Whole-genome sequencing of in vitro clonally expanded single cells	Aging	COSMIC signatures 1, 5, and 8
Zhang et al. (132)	B lymphocytes	Single-cell whole-genome sequencing	Aging	COSMIC signatures 1 and 5
Lee-Six et al. (70)	Colon (crypts)	Whole-genome sequencing of colorectal crypts, to represent clones from colorectal stem cells	Aging	COSMIC signatures SBS5 and SBS1
Franco et al. (36)	Kidney tubules, epidermis, subcutaneous adipose tissue, visceral adipose tissue	Whole-genome sequencing of in vitro clonally expanded single cells	Aging	COSMIC signatures SBS1, SBS3/8, SBS5, and SBS40

a Catalogue of Somatic Mutations in Cancer (COSMIC) v3 single-base substitution signatures SBS1 and SBS5 are similar and analogous to COSMIC v2 signatures 1 and 5, respectively (126).
